# Clusters of people with type 2 diabetes in the general population: unsupervised machine learning approach using national surveys in Latin America and the Caribbean

**DOI:** 10.1136/bmjdrc-2020-001889

**Published:** 2021-01-29

**Authors:** Rodrigo M Carrillo-Larco, Manuel Castillo-Cara, Cecilia Anza-Ramirez, Antonio Bernabé-Ortiz

**Affiliations:** 1 Department of Epidemiology and Biostatistics, School of Public Health, Imperial College London, London, UK; 2 CRONICAS Centre of Excellence in Chronic Diseases, Universidad Peruana Cayetano Heredia, Lima, Peru; 3 Universidad Católica Los Ángeles de Chimbote, Instituto de Investigación, Chimbote, Peru; 4 Center of Information and Communication Technologies, Universidad Nacional de Ingeniería, Lima, Peru; 5 Universidad Científica del Sur, Lima, Peru

**Keywords:** adult, developing countries, diabetes mellitus, type 2

## Abstract

**Introduction:**

We aimed to identify clusters of people with type 2 diabetes mellitus (T2DM) and to assess whether the frequency of these clusters was consistent across selected countries in Latin America and the Caribbean (LAC).

**Research design and methods:**

We analyzed 13 population-based national surveys in nine countries (n=8361). We used k-means to develop a clustering model; predictors were age, sex, body mass index (BMI), waist circumference (WC), systolic/diastolic blood pressure (SBP/DBP), and T2DM family history. The training data set included all surveys, and the clusters were then predicted in each country-year data set. We used Euclidean distance, elbow and silhouette plots to select the optimal number of clusters and described each cluster according to the underlying predictors (mean and proportions).

**Results:**

The optimal number of clusters was 4. Cluster 0 grouped more men and those with the highest mean SBP/DBP. Cluster 1 had the highest mean BMI and WC, as well as the largest proportion of T2DM family history. We observed the smallest values of all predictors in cluster 2. Cluster 3 had the highest mean age. When we reflected the four clusters in each country-year data set, a different distribution was observed. For example, cluster 3 was the most frequent in the training data set, and so it was in 7 out of 13 other country-year data sets.

**Conclusions:**

Using unsupervised machine learning algorithms, it was possible to cluster people with T2DM from the general population in LAC; clusters showed unique profiles that could be used to identify the underlying characteristics of the T2DM population in LAC.

**Significance of this study**

**What is already known about this subject?**

- Clustering analysis has been used to group patients with diabetes according to underlying factors and to assess the long-term outcomes of these groups; however, these works focused on reduced samples of patients and analyzed sophisticated predictors, limiting the applicability of these models to large population-based studies.

**What are the new findings?**

- We showed that large population-based surveys, along with unsupervised clustering analysis informed by simple predictors, could provide relevant groups of patients with type 2 diabetes mellitus in the general population.
- The four clusters were well characterized by one or few predictors; for example, the mean age was highest in cluster 3; the mean body mass index and waist circumference were highest in cluster 1; and systolic and diastolic blood pressure were highest in cluster 0.

**How might these results change the focus of research or clinical practice?**

- Our work borrows a methodology that previously was applied to groups of patients from limited clinical sites and was informed by sophisticated variables; in so doing, our work may spark interest to implement these analytical techniques in (large) populations, rather than focusing on individual patients.
- We delivered clusters of patients in the general population, which could help in monitoring the underlying factors of people with type 2 diabetes, thus informing interventions and policies aimed at the general population level.

## Introduction

Type 2 diabetes mellitus (T2DM) poses a large disease burden globally and in Latin America and the Caribbean (LAC), where there are two of the top 10 countries with the largest number of people with T2DM.^1–4^ T2DM also represents an economic burden to patients and health systems^5^ which do not have the resources to conduct mass screenings.^6^ Consequently, awareness about T2DM status is suboptimal in low-income and middle-income countries and LAC.^6^ For those who have been diagnosed with T2DM, there are effective non-pharmacological and pharmacological treatments;^7 8^ however, T2DM treatment coverage is also limited in LAC.^6 9^

People with long-term undiagnosed T2DM and those who cannot receive effective treatment are at high risk of T2DM-related complications and other unfavorable outcomes. Risk stratification tools are very helpful to identify patients at higher risk of specific outcomes.^10–13^ Cluster analysis using novel analytical techniques (eg, machine learning) has also proven to successfully stratify patients with T2DM and link these clusters to clinically important outcomes.^14–17^ However, clusters are usually based on sophisticated predictors that are not available in low-income and middle-income countries or large population-based epidemiological surveys. Whether these novel methods can be applied to population-based data using simple predictors while also classifying patients with T2DM in groups with similar profiles has not been studied. Identifying clusters in the general population and quantifying their frequency and trends could provide insights about the underlying characteristics of patients with T2DM in a population. Studying these clusters across time would provide evidence about changes in the underlying characteristics of the T2DM population. Finally, if countries in the same region do not consistently show the same cluster distribution, this may challenge the need for regional-based policies in favor of country-specific policies.

Previously available works focused on clustering sophisticated predictors in reduced samples of patients with T2DM;^14–17^ instead, we aimed to develop a clustering model for the general population based on simple predictors that are routinely available in large population-based surveys. This preliminary work will follow an unsupervised machine learning approach; for this, cross-sectional population-based national surveys in nine LAC countries were used to identify and quantify the frequency of clusters of patients with T2DM. We also aimed, exploratorily, to study whether the same cluster configuration applies to all selected countries. In this way, we will lay the foundations for the identification of potentially relevant T2DM groups in the general population in LAC. Overall, our research question is: if we developed clusters for people with T2DM in LAC, would the distribution of these clusters be consistent across countries?

## Research design and methods

### Data sources

We analyzed 13 country-year data sets. These are population-based national surveys in LAC that had at least one diabetes biomarker (eg, fasting glucose); that is, national surveys without blood samples or diabetes biomarkers were excluded. We pooled STEPwise approach to surveillance (STEPS) surveys^18–21^ and other surveys conducted by governmental bodies in each country.^22–26^ These surveys studied a random sample of the general population and followed standard procedures. Relevant variables were homogenized and pooled for this analysis (online supplemental table 1). These surveys can be downloaded and accessed online free of charge.

10.1136/bmjdrc-2020-001889.supp1 Supplementary data

### Study population

We only studied people with T2DM; in other words, we excluded people who did not have T2DM in each survey. We defined T2DM as any of the following: fasting glucose ≥126 mg/dL, self-reported diagnosis or receiving treatment for T2DM (online supplemental table 1).

### Machine learning analysis

Using an unsupervised machine learning approach and benefitting from population-based national surveys in LAC, we aimed to fit a classification (or clustering) model to identify and quantify the relative frequency of data-driven groups of people with T2DM. Overall, a global fit that consisted of merging all data sets of all country-years and then making a prediction for each data set (country-year) was performed. First, we pooled all data sets to run the fit analysis; that is, with the pooled data set we performed the dimensionality reduction (principal component analysis (PCA)) and the training phase of the k-means clustering algorithm. Second, we used this trained fit to predict in each country-year data set. We followed this procedure to observe local (each country-year) changes with respect to a global analysis (ie, identify clusters in each country-year data set with the model fitted having pooled all data sets). Variations in cluster distribution in each country-year with respect to the global fit inform about potential local differences. This way, we can see how the individual cluster in each country-year varies with respect to a global model. Similarly, variations across years for the same country inform of potential time trends. In both cases—local difference or time trend—our approach provides empirical preliminary evidence of potential differences and changes in the underlying profile of the population with T2DM in LAC.

### Data preparation for machine learning analysis

#### Predictors

We analyzed predictors of different nature. There were qualitative predictors coded as discrete variables, for example sex and family history of T2DM. There were also quantitative predictors such as body mass index (BMI, kg/m^2^), waist circumference (WC, cm), systolic blood pressure (SBP) and diastolic blood pressure (DBP), and for these we removed outliers by excluding observations at 5 SD below or above the mean.

Machine learning analysis requires that the variables are in the same scale. We tried different data transformation techniques, including scaling, standardizing and normalizing; however, an orthogonal transformation of variables accounting for the explained variability was the most robust technique. Among dimension reduction techniques and considering the explained variance, PCA was the one that validated the most robust clustering.

### Clustering machine learning analysis

#### Principal component analysis

PCA falls in the field of unsupervised machine learning algorithms. PCA follows an orthogonal transformation which turns correlated variables into an uncorrelated set of variables.^27^ The PCA aims to create a—reduced—set of characteristics or components that still carries the relevant information from the original group of variables. The authors have followed a similar approach in a previous clustering analysis at the country level.^28^

#### PCA and fit

Regarding the PCA parameters, we specified those that provided the most robust results in the clustering (k-means) analysis; these parameters were (1) *whiten=true*: to guarantee uncorrelated outputs with unit variations of the components; and (2) *svd_solver=‘auto’*: because the pooled data set was not of large dimensionality, the singular value decomposition (SVD) selected the components with the LAPACK algebraic method, which selects the components through a postprocessing transformation. The other parameters used for PCA were set to default values using the Scikit-Learn Python PCA decomposition library.^29^ Finally, three PCA components were selected because they explained 95% of the variance.

#### Transform

The PCA model, which was fitted with the pooled data set as specified above, was applied to each country-year data set. Thus, each country-year data set was orthogonally transformed based on what the PCA model had learned from the pooled data set. In this transformation phase, there were no parameter adjustment or explained variance because these were from the fitted PCA model trained with the pooled data set.

#### k-means

This was the model used to cluster people with T2DM in data-driven groups. This unsupervised machine learning technique assigns heterogeneous elements of a data set into homogeneous clusters which were unknown at the beginning of the analysis. As justified in a previous work,^28^ k-means is a centroid-based algorithm that performs well when clusters have a globular shape and these are of similar size and density. Given our aims and data sets, k-means was considered as the best option.^30^ For the development of the k-means clustering method, a training fit with all the data sets was implemented; later, we made the prediction to each of the country-year data sets. Finally, considering the optimal number of clusters as supported by the three methods described in the next section, we used the k-means algorithm to generate four clusters. The other internal k-means parameters were set by default using the Scikit-Learn Library in Python.^31^

### Performance metrics of the machine learning model

The data-driven model to cluster people with T2DM could have offered different numbers of groups (or clusters); we studied the following parameters to select the most robust model. After the application of our centroid-based algorithm (ie, k-means approach), justification of the number and type of clusters is a subjective (logical or expert knowledge) and objective (numerical) judgment.^32 33^ Regarding the objective justification, all analyses have been validated with different techniques to verify the optimal number of clusters. First, the dendrogram with Euclidean distances gave four clusters with very similar Euclidean distances in them (online supplemental figure 1). These values certify high intracluster and low intercluster similarity. Second, the elbow method also supported that four clusters were optimal (online supplemental figure 2). Third, the silhouette plot showed that the highest average silhouette score obtained was at four clusters (online supplemental figure 3). Fourth, we used the Jaccard coefficient to study the stability of the clusters;^34 35^ a coefficient close to 1 suggests that clusters were well defined.^35^ The Jaccard coefficient for the four clusters was 0.976, 0.976, 0.964, and 0.976, respectively (online supplemental table 2). Consequently, the Jaccard coefficient suggested our clusters were well defined. Regarding subjective justification, please refer to the Discussion section, where we have elaborated on potential explanations for the cluster configuration and distribution. The selection metrics are reported in the Research design and methods section to support the selection of the final model. In the Results section, we focused on the description of the clusters, their characteristics and frequency.

10.1136/bmjdrc-2020-001889.supp3 Supplementary data

10.1136/bmjdrc-2020-001889.supp4 Supplementary data

10.1136/bmjdrc-2020-001889.supp5 Supplementary data

10.1136/bmjdrc-2020-001889.supp2 Supplementary data

## Results

### Data sources

In a complete-case analysis, and after dropping outlier observations (≥5 SD), we analyzed 13 (n=8361) country-year national surveys in LAC, including only people with T2DM: Argentina (2018, n=1985), Barbados (2007, n=59), Chile (2003, n=366; 2010, n=495; 2017, n=888), Costa Rica (2005, n=516), El Salvador (2016, n=476), Mexico (2016, n=935; 2019, n=2045), Peru (2005, n=151), Uruguay (2006, n=62; 2014, n=252), and British Virgin Islands (2009, n=131).

### Clusters

**Figure 1:**
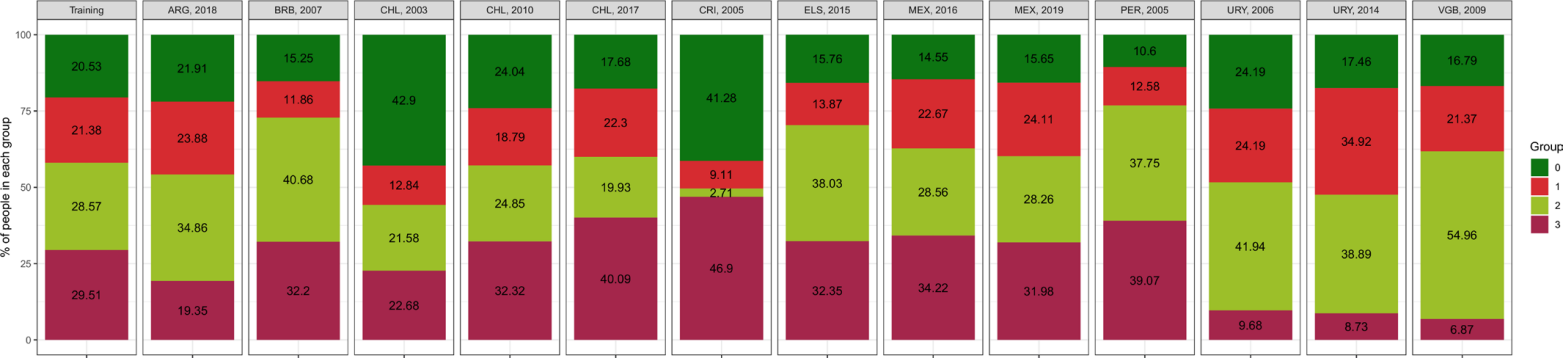
Cluster configuration in the training data set and in each country-year data set. ARG, Argentina; BRB, Barbados; CHL, Chile; CRI, Costa Rica; ELS, El Salvador; MEX, Mexico; PER, Peru; URY, Uruguay; VGB, British Virgin Islands.

The number of clusters with the best metrics was 4 (please refer to the Performance metrics of the machine learning model section). Observations in the training data set were classified almost evenly across the four clusters (figure 1): 20.5% in cluster 0, 21.4% in cluster 1, 28.6% in cluster 2 and 29.5% in cluster 3.

Cluster 0 outranked the other clusters, with the highest mean SBP, DBP and proportion of men (table 1). Cluster 1 outranked the others in three categories, showing the highest mean BMI, WC and proportion of relatives with diabetes (table 1). Cluster 2 stood out with the smallest mean values for almost all predictors: BMI, WC, SBP and men proportion (table 1). Cluster 3 only ranked first in one category: highest mean age (table 1).

**Table 1:** Characteristics of the clusters in the training data sets

	Age (years)	BMI (kg/m^2^)	WC (cm)	SBP (mm Hg)	DBP (mm Hg)	Men (%)	Diabetes in family (%)
Cluster 0	64.3 (10.1)	29.5 (4.6)	98.3 (10.4)	170.1 (18.2)	92.7 (12.1)	41.7	56.0
Cluster 1	52.9 (10.7)	37.0 (5.3)	116.6 (10.2)	131.7 (16.4)	83.1 (10.9)	38.6	64.2
Cluster 2	46.5 (8.9)	27.6 (4.0)	90.5 (9.3)	122.8 (13.7)	79.3 (10.0)	31.5	59.6
Cluster 3	70.4 (8.5)	28.5 (4.3)	97.6 (9.9)	131.0 (15.8)	71.3 (9.2)	36.7	54.5

A graphical representation of these summaries (eg, boxplots) is shown in online supplemental figure 5.

Summaries for numeric variables (age, BMI, WC, SBP and DBP) are shown as mean along with SD.

Red shade for the largest mean, blue for the second largest, yellow for the third largest and green for the smallest value within each column.

BMI, body mass index; DBP, diastolic blood pressure; SBP, systolic blood pressure; WC, waist circumference.

10.1136/bmjdrc-2020-001889.supp7 Supplementary data

When we reflected these four clusters in each country-year data set, a different distribution of each cluster was observed (figure 1). As it was the case in the training data set, cluster 3 had the largest proportion in 7 (out of 13) country-year data sets; conversely, cluster 3 became the least frequent in four country-year data sets. Cluster 0 was the least frequent in the training data set, and so it was in five other country-year data sets; conversely, cluster 0 became the most frequent in two country-year data sets (figure 1).

The largest shrinkage was observed in cluster 2, the frequency of which decreased from 28.6% in the training data set to 2.7% in one country-year data set. Cluster 2 also experienced the largest increase, moving from 28.6% to 55.0% (figure 1).

For countries with more than 1 year of available data, some clusters were consistent, yet others changed in time. Mexico contributed to the analysis with 2 years and clusters were largely consistent (figure 1). For Uruguay we also had 2 years and we observed a change: the frequency of cluster 0 was 24.2% in the first year, whereas it was 17.5% in the second year; similarly, 24.2% of the population were in cluster 1 in the first year, while 34.9% were in this cluster in the second year (figure 1). We analyzed 3 years of data for Chile. There was an increasing trend for cluster 3: 22.7%, 32.3% and 40.1%; there was also an increasing trend for cluster 1: 12.8%, 18.8% and 22.3%. Conversely, the frequency for cluster 0 decreased: 42.9%, 24.0% and 17.7% (figure 1).

## Discussion

### Main results

We developed an unsupervised machine learning model to cluster people with T2DM from the general population in nine countries in LAC. The optimal number of clusters was 4, each with unique features. One cluster grouped a higher proportion of men and those with high blood pressure; other clusters included people with high BMI, WC as well as high frequency of relatives with diabetes. The frequency of the clusters was not always consistent across country-year data sets. The cluster profile could reveal underlying risk factors in people with T2DM in the general population; patients in different clusters could need tailored management and prevention. Changes across time and countries could also reveal variations in the underlying risk factor profile in the population or changes in the health system capacity, for example better diagnosis and treatment coverage. We used machine learning methods previously applied only to individuals and not to large populations,^14–17^ thus advancing the field with a preliminary work that sets the foundations for the identification of potentially relevant T2DM groups in the general population in LAC benefitting from population-based national surveys.

### Potential explanations and implications

A mechanistic understanding of the etiology of each cluster is beyond the scope of this work (ie, this is not etiological research); conversely, we aimed to identify and quantify data-driven clusters of patients with T2DM in the general population in LAC. However, we discuss the profile of each cluster, relate it to T2DM key features, and propose possible applications of these clusters.

Cluster 0 and cluster 1 could represent two different groups of patients. Cluster 0 grouped most men and those with the highest mean blood pressure. People in this group could be at higher risk of cardiovascular events (eg, myocardial infarction or stroke) and may benefit from treatment or interventions to reduce and control blood pressure and possibly other associated conditions (eg, dyslipidemia). On the other hand, cluster 1 groups three variables that are the cornerstone of T2DM diagnosis and prediction: high body weight and relatives with diabetes. People in this cluster could be at higher risk of not achieving optimal metabolic control^36 37^ or of T2DM-related complications.^38^ People in cluster 1 could benefit from thorough metabolic control, with weight reduction and close medication monitoring. Other hypotheses may imply time elapsed from diagnosis. If patients with a recent diagnosis were included in cluster 1, then T2DM would not have yet caused weight loss, unlike patients who have long-lasting undiagnosed and/or uncontrolled T2DM. Regardless of time of diagnosis, metabolic and weight control could be key interventions for people in this cluster.

Cluster 2 showed the smallest or the second to the last smallest levels in all predictors, except in relatives with diabetes. Patients in this group are likely to have controlled T2DM, with weight and blood pressure apparently in optimal ranges. Nonetheless, they had the second largest frequency of relatives with diabetes. People in this group could probably benefit from family-based interventions.^39 40^ This could be beneficial for them, but also for other family members who have not been diagnosed with T2DM, for whom T2DM could be delayed or prevented.

Cluster 3 showed all predictors almost in the middle of the distribution, except for age which mean was the highest in this cluster. People in this cluster are, perhaps, most likely to have had T2DM for a long time. They may have learned how to live responsibly with this disease while taking care of other concomitant conditions, such as weight control and blood pressure. They could benefit from regular check-ups to keep surveillance on medication and other risk factors.^41^

There seemed to be a heterogenous distribution of clusters across countries; that is, the characteristics and frequency of clusters were not identical between countries. This could signal different profiles of people with T2DM in these countries (eg, metabolic control) and different distributions of underlying risk factors (eg, obesity).^3^ Health system performance to prevent, diagnose and control T2DM could also potentially explain this finding. Nonetheless, we ought to keep in mind a key difference among the analyzed data sets: the age structure. This does not make all samples comparable. We did not restrict to a common age subset because we aimed to develop a clustering model that can use the full power of national population-based surveys, which are conducted periodically and with a consistent methodology; had we limited the study to the same age range, we would have lost sample size and included fewer countries, limiting the scope of our work.

This work has successfully classified people with T2DM in four clusters at the general population level in selected LAC countries. We have proposed a hypothesis to explain the cluster configuration; based on these assumptions, we have also proposed interventions for each cluster. Nonetheless, given the study design (cross-sectional data analysis), it is impossible to study the long-term outcomes of the identified clusters. It is also impossible to study whether the proposed interventions for each cluster would have a positive effect on clinical outcomes. Future work, ideally multicountry cohorts in LAC, would need to elaborate on our work and study long-term outcomes. Currently, we provide data-driven clusters useful to identify groups of patients with T2DM in the general population in LAC, but this deserves further prospective research.

### Public health implications

This work analyzed population-based surveys to provide an overall picture of clusters of people with T2DM at the country level in LAC. In so doing, we found that it would be optimal to classify patients with T2DM in four clusters. We also found that the proportion of each cluster is not consistent across countries and years. This suggests that the characteristics of people with T2DM do not distribute equally in the selected countries.^3^ This has implications for regional and national interventions. First, regional guidelines and recommendations should secure that, when relevant and possible, interventions are tailored or can be adapted to the reality or profile of each country. The cluster patterns herein depicted suggest that people with T2DM do not always have the same underlying risk factor levels across countries and time. Second, when countries adopt successful T2DM interventions from other countries, careful consideration is warranted to assess whether tailoring or adaptation is needed. It may be possible that a successful intervention in one country does not have the same impact in another, if the underlying profile of the patients is different. Finally, interventions should not be static and periodic assessment is needed to understand if the population still shows the same profiles, or conversely they need a new or updated intervention.

We provided a potential tool for surveillance of groups of people with T2DM in the general population. If we have four clusters and in 2015, 30% of the T2DM population in country X belonged to cluster 0, 20% to cluster 1, 40% to cluster 2, and 10% to cluster 3. This would give an idea of the overall underlying profile of people with T2DM. If cluster 2 was characterized by poor metabolic control, then we would need to improve this (eg, securing treatment). If we repeat the analysis in 2020, the frequency of these clusters could change to the following: cluster 0 with 70%, cluster 1 with 15%, cluster 2 with 10% and cluster 3 with 5%. Then, this would give evidence that in the last 5 years something in the population has changed (this would need further investigation), because the T2DM population is now -in 2020- pronominally in cluster 0. If cluster 0 was characterized by high obesity rates, then this would suggest that we need to secure better weight management or introduce other food policies.

### Strengths and limitations

We used national surveys, which account for an overall good representation of the general population in the selected LAC countries. Our inclusion criteria for T2DM accounted for known and unknown T2DM, providing evidence for the overall T2DM population while maximizing the study sample. However, limitations need to be acknowledged too. First, despite using national surveys, we analyzed a small sample size. This was because we only studied people with T2DM, rather than the whole population. Our results could be verified with a larger sample of people with T2DM, although it is unlikely to find such sample with a national scope. Second, T2DM status was based on self-reported diagnosis or glucose tests. It could be argued that other biomarkers (eg, hemoglobin A1c (HbA1c)) could provide better diagnosis or could be complementary. Conducting more sophisticated blood tests is challenging, not to mention expensive, in large random population-based samples, particularly in national surveys. Even if other tests were available, we would have diagnosed few more cases; we argue that this would not have substantially changed our results. Third, other variables, for example HbA1c or microvascular/macrovascular complications, were not available in all national surveys, so further analysis by metabolic control or T2DM-related complications could not be conducted. Nonetheless, this limitation further supports our argument to use simple variables to provide evidence for the general population, and not only more sophisticated markers that may not be always available in large national surveys, which can inform public health interventions. Fourth, the surveys we analyzed had different age structures; for example, some studied people younger than 65 years and others included older individuals. In that sense, comparisons across countries need to be made cautiously and consider these differences. We did not restrict the samples to the same age range because (1) we aimed to maximize sample size and the number of available surveys, hence the number of countries; and (2) we aimed to develop a model that would benefit from, and could be applied to, available national surveys which are conducted periodically and following the same methodology. If we had developed a model for a subsample, then the full power of a national data set would not have been used. Data maximization is paramount for machine learning research. Fifth, we did not compare our models with others available in the literature.^14–17^ A head-to-head comparison with other models was beyond the scope of this work because we targeted a different population and had a different rationale for our work. Previous models aimed to precisely classify individual patients based on clinical or sophisticated predictors and to understand what outcomes they were most likely to experience.^14–17^ Conversely, we targeted the general population and aimed to identify clusters of patients, from the general population, based on simple variables that are available in large national surveys (eg, weight, height and blood pressure). Available models have a strong place in clinical medicine, while we hope our work can inform population health efforts to identify, quantify and monitor clusters of patients with T2DM in the general population. Sixth, because of the nature of the data herein analyzed—national population-based surveys—we could not look into long-term outcomes, like studies with a reduced sample and more sophisticated predictors have done.^14–17^ Our work complement this stronger evidence by suggesting that machine learning clustering analysis could also provide relevant information applied to larger national surveys. Future work should elaborate on the long-term outcomes based on the clusters herein developed. Seventh, the analyzed data were collected in different years, which could have introduced time bias. We do not consider this a serious limitation to our results because we aimed to provide a broad picture for the region in a wide (~10 years) time frame. Eight, because we converged several data sets from countries in LAC, we believe our models could be applied to other countries not herein studied; however, extrapolation to other world regions would require further verification and cautious interpretation. Eighth, although we acknowledge that sex is an important variable in T2DM epidemiology, our clustering model included sex as a predictor rather than conducting the analysis stratified by sex. However, when we verified our clusters stratified by sex (online supplemental figure 4), the cluster configuration and the relative proportion of each cluster were very similar between men and women, as well as in comparison with the overall results herein presented. Small noted differences are most likely due to a misbalance of sex.

10.1136/bmjdrc-2020-001889.supp6 Supplementary data

## Conclusions

An unsupervised machine learning approach to cluster people with T2DM in the general population of selected LAC countries revealed groups with unique features. These clusters could be used for risk stratification and to propose interventions or policies for different countries in LAC to reduce T2DM burden based on the underlying profile of people with T2DM. The clusters revealed that this profile is not identical across countries, and even within countries these clusters may change over the years. Meaningful short-term, mid-term and long-term associations of these clusters warrant further investigation.

## References

[1] GBD 2017 Risk Factor Collaborators Global, regional, and national comparative risk assessment of 84 behavioural, environmental and occupational, and metabolic risks or clusters of risks for 195 countries and territories, 1990-2017: a systematic analysis for the global burden of disease study 2017. Lancet 2018;392:1923–94. 10.1016/S0140-6736(18)32225-6 30496105 PMC6227755

[2] GBD 2017 Causes of Death Collaborators Global, regional, and national age-sex-specific mortality for 282 causes of death in 195 countries and territories, 1980-2017: a systematic analysis for the global burden of disease study 2017. Lancet 2018;392:1736–88. 10.1016/S0140-6736(18)32203-7 30496103 PMC6227606

[3] NCD Risk Factor Collaboration (NCD-RisC)—Americas Working Group Trends in cardiometabolic risk factors in the Americas between 1980 and 2014: a pooled analysis of population-based surveys. Lancet Glob Health 2020;8:e123–33. 10.1016/S2214-109X(19)30484-X 31839128 PMC7025323

[4] NCD Risk Factor Collaboration (NCD-RisC) Worldwide trends in diabetes since 1980: a pooled analysis of 751 population-based studies with 4.4 million participants. Lancet 2016;387:1513–30. 10.1016/S0140-6736(16)00618-8 27061677 PMC5081106

[5] Bommer C, Sagalova V, Heesemann E, et al Global economic burden of diabetes in adults: projections from 2015 to 2030. Diabetes Care 2018;41:963–70. 10.2337/dc17-1962 29475843

[6] Manne-Goehler J, Geldsetzer P, Agoudavi K, et al Health system performance for people with diabetes in 28 low- and middle-income countries: a cross-sectional study of nationally representative surveys. PLoS Med 2019;16:e1002751. 10.1371/journal.pmed.1002751 30822339 PMC6396901

[7] American Diabetes Association 9. Pharmacologic Approaches to Glycemic Treatment: Standards of Medical Care in Diabetes-2020. Diabetes Care 2020;43:S98–110. 10.2337/dc20-S009 31862752

[8] Raveendran AV, Chacko EC, Pappachan JM Non-Pharmacological treatment options in the management of diabetes mellitus. Eur Endocrinol 2018;14:31–9. 10.17925/EE.2018.14.2.31 30349592 PMC6182920

[9] Chow CK, Ramasundarahettige C, Hu W, et al Availability and affordability of essential medicines for diabetes across high-income, middle-income, and low-income countries: a prospective epidemiological study. Lancet Diabetes Endocrinol 2018;6:798–808. 10.1016/S2213-8587(18)30233-X 30170949

[10] Crawford F, Cezard G, Chappell FM, et al A systematic review and individual patient data meta-analysis of prognostic factors for foot ulceration in people with diabetes: the International research collaboration for the prediction of diabetic foot ulcerations (PODUS). Health Technol Assess 2015;19:1–210. 10.3310/hta19570 PMC4781379 26211920

[11] van der Heijden AA, Nijpels G, Badloe F, et al Prediction models for development of retinopathy in people with type 2 diabetes: systematic review and external validation in a Dutch primary care setting. Diabetologia 2020;63:1110–9. 10.1007/s00125-020-05134-3 32246157 PMC7228897

[12] van Dieren S, Beulens JWJ, Kengne AP, et al Prediction models for the risk of cardiovascular disease in patients with type 2 diabetes: a systematic review. Heart 2012;98:360–9. 10.1136/heartjnl-2011-300734 22184101

[13] Wang Y, Negishi T, Negishi K, et al Prediction of heart failure in patients with type 2 diabetes mellitus- a systematic review and meta-analysis. Diabetes Res Clin Pract 2015;108:55–66. 10.1016/j.diabres.2015.01.011 25686509

[14] Ahlqvist E, Storm P, Käräjämäki A, et al Novel subgroups of adult-onset diabetes and their association with outcomes: a data-driven cluster analysis of six variables. Lancet Diabetes Endocrinol 2018;6:361–9. 10.1016/S2213-8587(18)30051-2 29503172

[15] Dennis JM, Shields BM, Henley WE, et al Disease progression and treatment response in data-driven subgroups of type 2 diabetes compared with models based on simple clinical features: an analysis using clinical trial data. Lancet Diabetes Endocrinol 2019;7:442–51. 10.1016/S2213-8587(19)30087-7 31047901 PMC6520497

[16] Zaharia OP, Strassburger K, Strom A, et al Risk of diabetes-associated diseases in subgroups of patients with recent-onset diabetes: a 5-year follow-up study. Lancet Diabetes Endocrinol 2019;7:684–94. 10.1016/S2213-8587(19)30187-1 31345776

[17] Bello-Chavolla OY, Bahena-López JP, Vargas-Vázquez A, et al Clinical characterization of data-driven diabetes subgroups in Mexicans using a reproducible machine learning approach. BMJ Open Diabetes Res Care 2020;8:e001550. 10.1136/bmjdrc-2020-001550 PMC7380860 32699108

[18] World Health Organization NCD microdata repository. Barbados steps 2007, 2007 Available: https://extranet.who.int/ncdsmicrodata/index.php/catalog/612

[19] World Health Organization NCD microdata repository. Uruguay steps 2006, 2006 Available: https://extranet.who.int/ncdsmicrodata/index.php/catalog/734

[20] World Health Organization NCD microdata repository. Uruguay steps 2013, 2013 Available: https://extranet.who.int/ncdsmicrodata/index.php/catalog/628/

[21] World Health Organization NCD Microdata repository. British virgin Slands steps 2009, 2009 Available: https://extranet.who.int/ncdsmicrodata/index.php/catalog/613

[22] Instituto Nacional de Estadistica y Censos Republica de argentina. 4 encuesta nacional de factores de riesgo, 2020 Available: https://www.indec.gob.ar/indec/web/Nivel4-Tema-4-32-68

[23] Departamento de Epidmeiologia, Ministerio de Salud, Gobierno de Chile Encuesta nacional de salud, 2020 Available: http://epi.minsal.cl/bases-de-datos/

[24] CRELES Costa Rican study on longevity and healthy aging, 2020 Available: http://creles-download.demog.berkeley.edu/CRdata.pl

[25] El Salvador Encuesta nacional de Enfermedades cronicas, 2020 Available: https://data.amerigeoss.org/es/dataset/encuesta-nacional-de-enfermedades-cronicas

[26] Instituto Nacional de Salud Publica Mexcio. Encuesta nacional de salud Y nutricion, 2020 Available: https://ensanut.insp.mx/index.php 10.21149/17295 41150976

[27] Abdi H, Williams LJ Principal component analysis. Wiley Interdiscip Rev Comput Stat 2010;2:433–59. 10.1002/wics.101

[28] Carrillo-Larco RM, Castillo-Cara M Using country-level variables to classify countries according to the number of confirmed COVID-19 cases: an unsupervised machine learning approach. Wellcome Open Res 2020;5:56. 10.12688/wellcomeopenres.15819.3 32587900 PMC7308996

[29] sklearn Scikit learn: sklearn.decomposition. PCA, 2020 Available: https://scikit-learn.org/stable/modules/generated/sklearn.decomposition.PCA.html

[30] Kanungo T, Mount DM, Netanyahu NS, et al An efficient k-means clustering algorithm: analysis and implementation. IEEE Trans Pattern Anal Mach Intell 2002;24:881–92. 10.1109/TPAMI.2002.1017616

[31] sklearn Scikit learn: sklearn.cluster.Kmeans, 2020 Available: https://scikit-learn.org/stable/modules/generated/sklearn.cluster.KMeans.html

[32] Chang C-H, Ding Z-K Categorical data visualization and clustering using subjective factors. Data Knowl Eng 2005;53:243–62. 10.1016/j.datak.2004.09.001

[33] Anandarajan M, Hill C, Nolan T Practical text analytics. maximizing the value of text data advances in analytics and data science. Vol. 2. Chapter 7. Berlin: Springer, 2019.

[34] Hennig C Cluster-wise assessment of cluster stability. Comput Stat Data Anal 2007;52:258–71. 10.1016/j.csda.2006.11.025

[35] Kulma K Cluster validation in unsupervised machine learning, 2017 Available: https://kkulma.github.io/2017-05-10-cluster-validation-in-unsupervised-machine-learning/

[36] Brown SA, García AA, Brown A, et al Biobehavioral determinants of glycemic control in type 2 diabetes: a systematic review and meta-analysis. Patient Educ Couns 2016;99:1558–67. 10.1016/j.pec.2016.03.020 27036083 PMC5028237

[37] Yaghoubi M, Mansell K, Vatanparastc H, et al Effects of Pharmacy-Based interventions on the control and management of diabetes in adults: a systematic review and meta-analysis. Can J Diabetes 2017;41:628–41. 10.1016/j.jcjd.2017.09.014 29224636

[38] Tracey ML, McHugh SM, Fitzgerald AP, et al Risk factors for macro- and microvascular complications among older adults with diagnosed type 2 diabetes: findings from the Irish longitudinal study on ageing. J Diabetes Res 2016;2016:1–9. 10.1155/2016/5975903 PMC4884580 27294152

[39] Torenholt R, Schwennesen N, Willaing I Lost in translation--the role of family in interventions among adults with diabetes: a systematic review. Diabet Med 2014;31:15–23. 10.1111/dme.12290 23870045

[40] Armour TA, Norris SL, Jack L, et al The effectiveness of family interventions in people with diabetes mellitus: a systematic review. Diabet Med 2005;22:1295–305. 10.1111/j.1464-5491.2005.01618.x 16176186

[41] Mehdi Hazavehei SM, Khoshravesh S, Taheri-Kharameh Z Increasing medical adherence in elderly with type 2 diabetes mellitus: a systematic review. Int Q Community Health Educ 2019;39:109–17. 10.1177/0272684X18819969 30799762

