# Supplementary material for: Clusters of people with type 2 diabetes in the general population: unsupervised machine learning approach using national surveys in Latin America and the Caribbean
Source: BMJ Open Diabetes Res Care. 2021 Jan 29;9(1):e001889. doi: 10.1136/bmjdrc-2020-001889 (PMC7849890; doi:10.1136/bmjdrc-2020-001889)
Supplement: Supplementary data [file bmjdrc-2020-001889supp001.pdf]

Supplementary Table 1: Characteristics of data sources analysed

Survey information					Measured (M) or Self-reported(S)			Diabetes				
									Diabetes biomarker			
**Survey**	**Country**	**Year (s)**	**Population**	**Selection of respondent**	**BMI**	**WC**	**SBP / DBP**	**Family history of Diabetes**	**Definition**	**Fasting (Time if available)**	**Method**	**Comment**
National Risk Factors Survey (ENFR 2018)	Argentina	2018	Urban male and female residents ≥ 18 years old	One individual randomly selected in each household (75% of the whole sample)	M	M	M	¿Has been at least a diabetes diagnosis between your blood relatives (parents, sons, daughters, siblings)?	Fasting glycaemia ≥126 mg/dL or self-reported diagnosed DM or medication	Yes	POC capillary glucose testing	If insulin-dependent, sample was taken before insulin injection
STEPS Barbados	Barbados	2007	Men and women aged 25-64 years	One individual randomly selected in each household (overall response rate: 65%)	M	M	M	Have some of your family members been diagnosed with the following diseases? Diabetes	FPG ≥126 mg/dL or self-reported doctor diagnosed DM or medication	Yes (8 - 12 hrs)	Laboratory testing: glucose hexokinase method	If participant was not fasting and no other appointment could be made, the sample was collected and the non-fasting status, recorded
National Health Survey (ENS 2003)	Chile	2003	Men and women ≥ 17 years old	Subject previously participated in the Quality of Life Survey in 2000	M	M	M	What other type of family health history do you have? Father with diabetes, Mother with diabetes	FPG ≥126 mg/dL or self-reported diagnosed DM or medication	Yes (≥ 8 hrs)	Laboratory testing: glucose hexokinase method	
National Health Survey (ENS 2010)	Chile	2010	Men and women ≥ 15 years old	Individuals ≥ 65 years old had twice the probability to be selected. Only one individual per household	M	M	M	In your immediate family (son, daughter, dad, mom or siblings), has anyone ever gotten sick or died of ...? Diabetes	FPG ≥126 mg/dL or self-reported diagnosed DM or medication	Yes (≥ 8 hrs)	Laboratory testing: glucose hexokinase method	Does not include gestational diabetes
National Health Survey (ENS 2017)	Chile	2017	Men and women ≥ 15 years old	Individuals ≥ 65 years old had twice the probability to be selected. Only one individual per household	M	M	M	In your immediate family: Has anyone gotten sick or died from Diabetes (high blood sugar)?	FPG ≥126 mg/dL or self-reported diagnosed DM or medication	Yes (≥ 8 hrs)	Laboratory testing: glucose hexokinase method	Does not include gestational diabetes

Carrillo-Larco RM, *et al. BMJ Open Diab Res Care* 2021; 9:e001889. doi: 10.1136/bmjdrc-2020-001889

Costa Rican Longevity and Healthy Aging Study (CRELES)	Costa Rica	2005	Men and women ≥ 60 years old	Oversampling of the oldest old	M	M	M	Is there anyone in your family who suffers or suffered from diabetes? parents, siblings, grandparents?	FPG ≥126mg/dL or self-report diagnosed DM or medication	Yes (14 hrs)	Laboratory testing: an enzymatic method for glucose	
Survey of Chronic Noncommunicable Diseases in Adults in El Salvador (ENECA-ELS)	El Salvador	2015	Men and women ≥ 20 years old	All individuals corresponding to the target population residing in selected households were included in the sample	M	M	M	Do any of your relatives (mother, father) have diabetes?	FPG ≥126 mg/dL or self-report diagnosed DM or medication	Yes (8 hrs)	Laboratory testing: glucose hexokinase method	
National Survey of Nutritional, Biochemical, Socioeconomic and Cultural Indicators Related to Chronic Degenerative Diseases	Peru	2005	Men and women ≥ 20 years old	No pregnant women as well as subjects who practice bodybuilding and qualified athletes were included	M	M	M	Do you have or have you had blood relatives with glucose problems?	FPG ≥126 mg/dL or self-report diagnosed DM or medication	Yes	Laboratory testing: Trinder-glucose oxidase-peroxidase method	
Halfway National Health and Nutrition Survey (ENSANUT-MC)	Mexico	2016	Men and women ≥ 20 years old	One individual randomly selected in each household (60% of the whole sample)	M	M	M	Does your father or mother have or had diabetes or high blood sugar?	FPG ≥126mg/dL or self-report diagnosed DM or medication	Yes (≥ 8 hrs)	Laboratory testing: glucose oxidase method	Does not include gestational diabetes
National Health and Nutrition Survey (ENSANUT 2018)	Mexico	2018	Men and women ≥ 20 years old	The number of selected individuals per household depends on the household size	M	M	M	Now, I will ask you about some diseases that your parents or siblings may have had: Diabetes	FPG ≥126mg/dL or self-report diagnosed DM or medication	Yes (≥ 8 hrs)	Laboratory testing: glucose oxidase method	There is a question for gestational diabetes
STEPS Uruguay	Uruguay	2006	Men and women aged 25-64 years	One individual randomly selected in each household (response rate: 33.7%)	M	M	M	Have some of your family members been diagnosed with the following diseases?	FPG ≥126 mg/dL or self-reported diagnosed DM or medication	Yes (8 - 12 hrs)	Laboratory testing: glucose hexokinase method	If participant was not fasting and no other appointment could be made, the sample was collected and the non-fasting status, recorded

STEPS Uruguay	Uruguay	2014	Urban male and female residents aged 15-64 years	One individual randomly selected in each household (response rate: 54.1%)	M	M	M	Have some of your family members been diagnosed with the following diseases?	Fasting glycemia ≥126 mg/dL or self-reported diagnosed DM or medication	Yes	POC capillary glucose testing	If participant was not fasting and no other appointment could be made, the sample was collected and the non-fasting status, recorded
STEPS British Virgin Islands	British Virgin Islands	2009	Men and women aged 25-64 years	One individual randomly selected in each household (25%). Pregnant women were not included.	M	M	M	Have some of your family members been diagnosed with the following diseases?	Capillary whole blood value: 26.1 mmol/L (l l0 mg/dl) or medication	Yes (8 - 12 hrs)	POC capillary glucose testing	

BMI: body mass index; WC: waist circumference; SBP: systolic blood pressure; DBP: diastolic blood pressure; STEPS: STEPwise Surveillance; FPG: Fasting plasma glucose; DM: Diabetes mellitus; HbA1c: glycosylated haemoglobin; POC: Point-of-care; HPLC: high-performance liquid chromatographic
