# Supplementary figures and images for: Clusters of people with type 2 diabetes in the general population: unsupervised machine learning approach using national surveys in Latin America and the Caribbean
Source: BMJ Open Diabetes Res Care. 2021 Jan 29;9(1):e001889. doi: 10.1136/bmjdrc-2020-001889 (PMC7849890; doi:10.1136/bmjdrc-2020-001889)

**Supplementary Figure 1: Dendrogram analysis**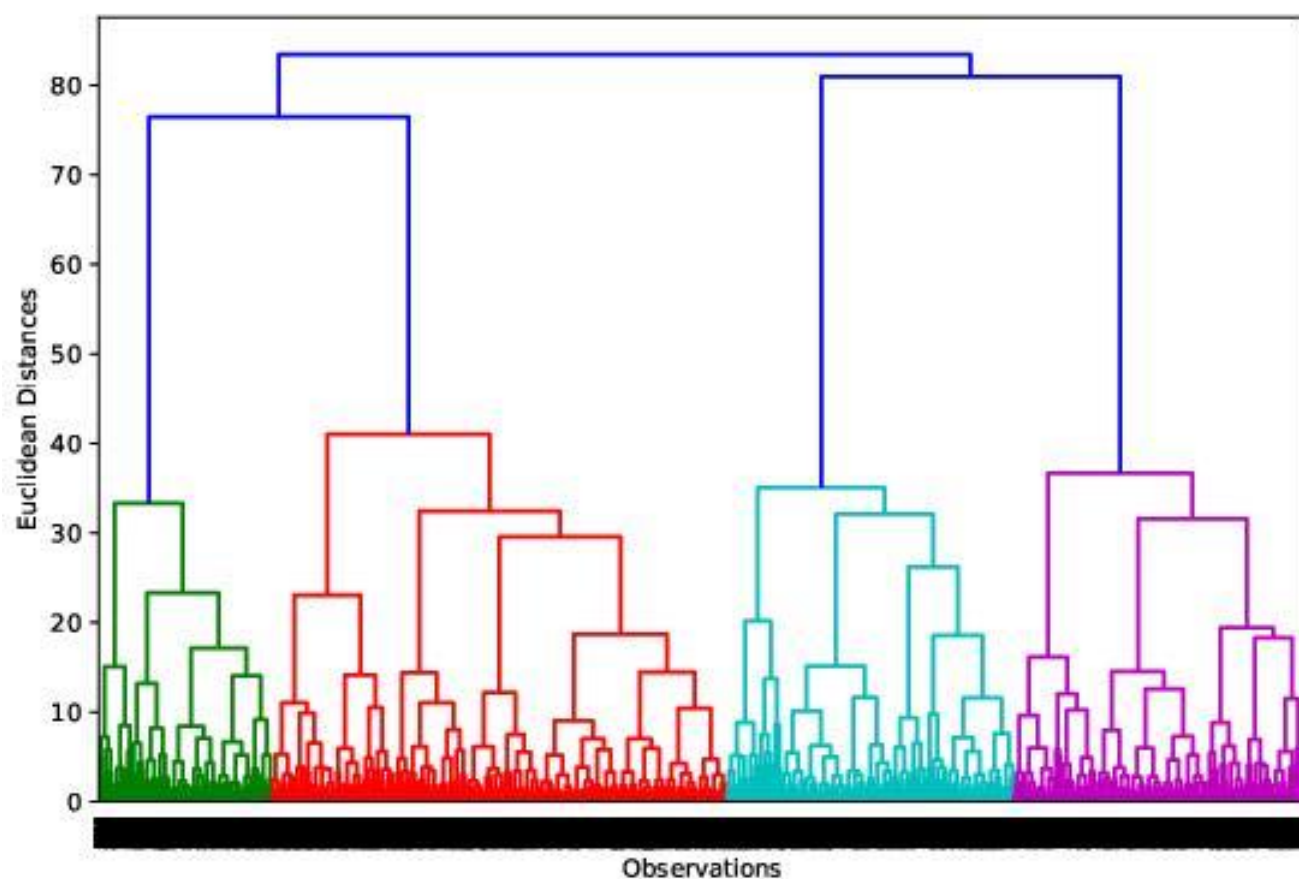

Supplement: Supplementary data [file bmjdrc-2020-001889supp003.pdf]

**Supplementary Figure 2: Elbow plot**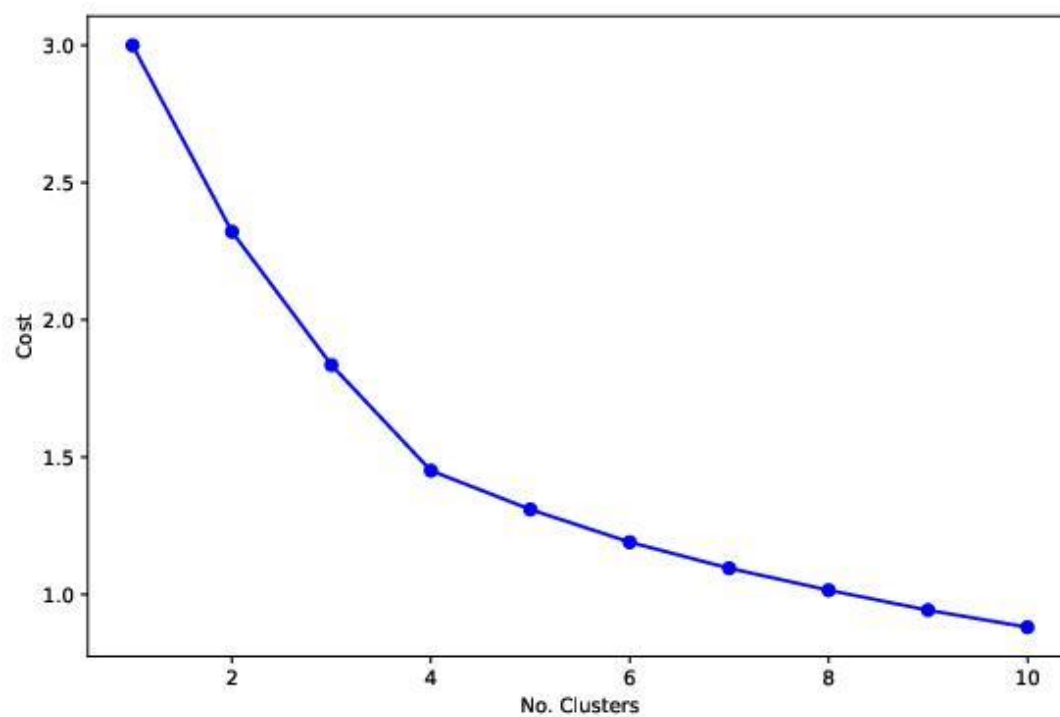

Supplement: Supplementary data [file bmjdrc-2020-001889supp004.pdf]

Supplementary Figure 3: Silhouette plot

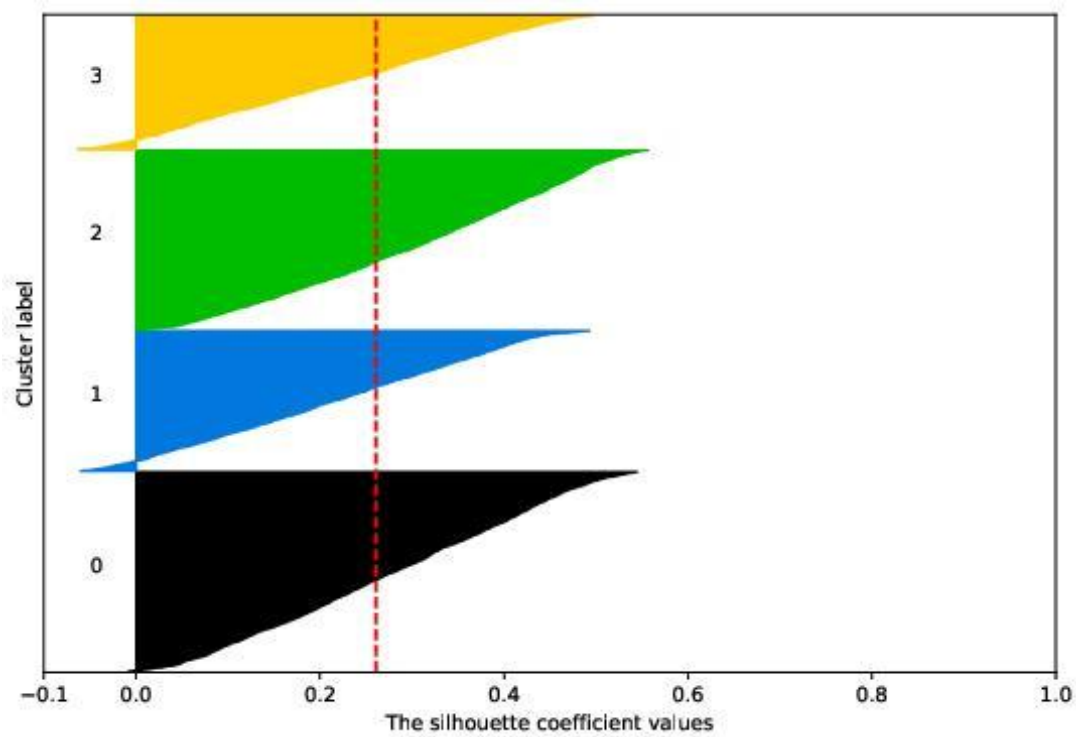

Supplement: Supplementary data [file bmjdrc-2020-001889supp005.pdf]

**Supplementary Figure 5: Distribution and frequency of predictors by analysed survey**

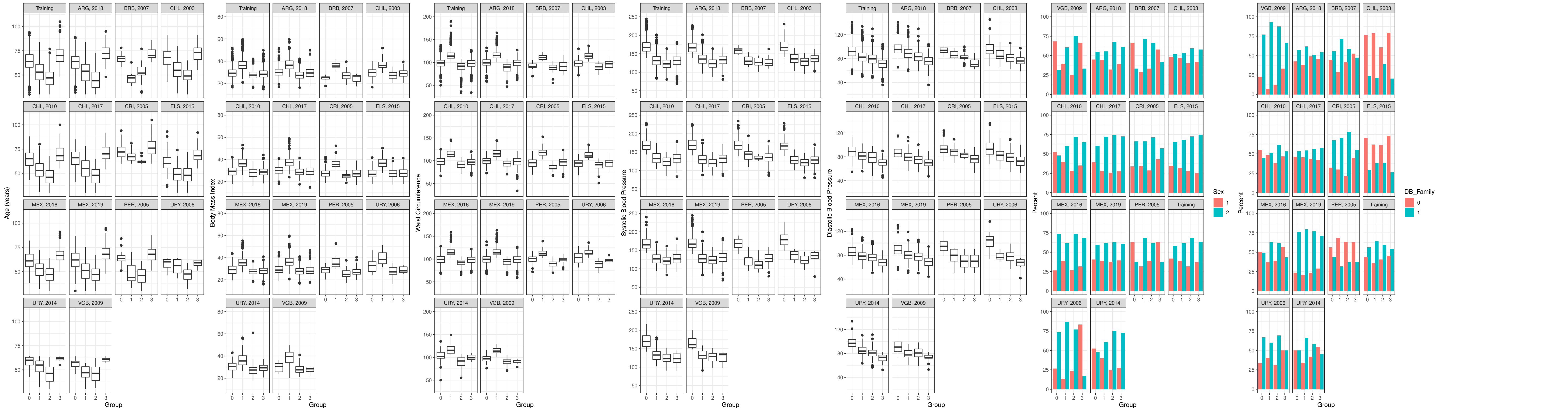

Supplement: Supplementary data [file bmjdrc-2020-001889supp007.pdf]

Supplementary Figure 4: Cluster analysis by gender

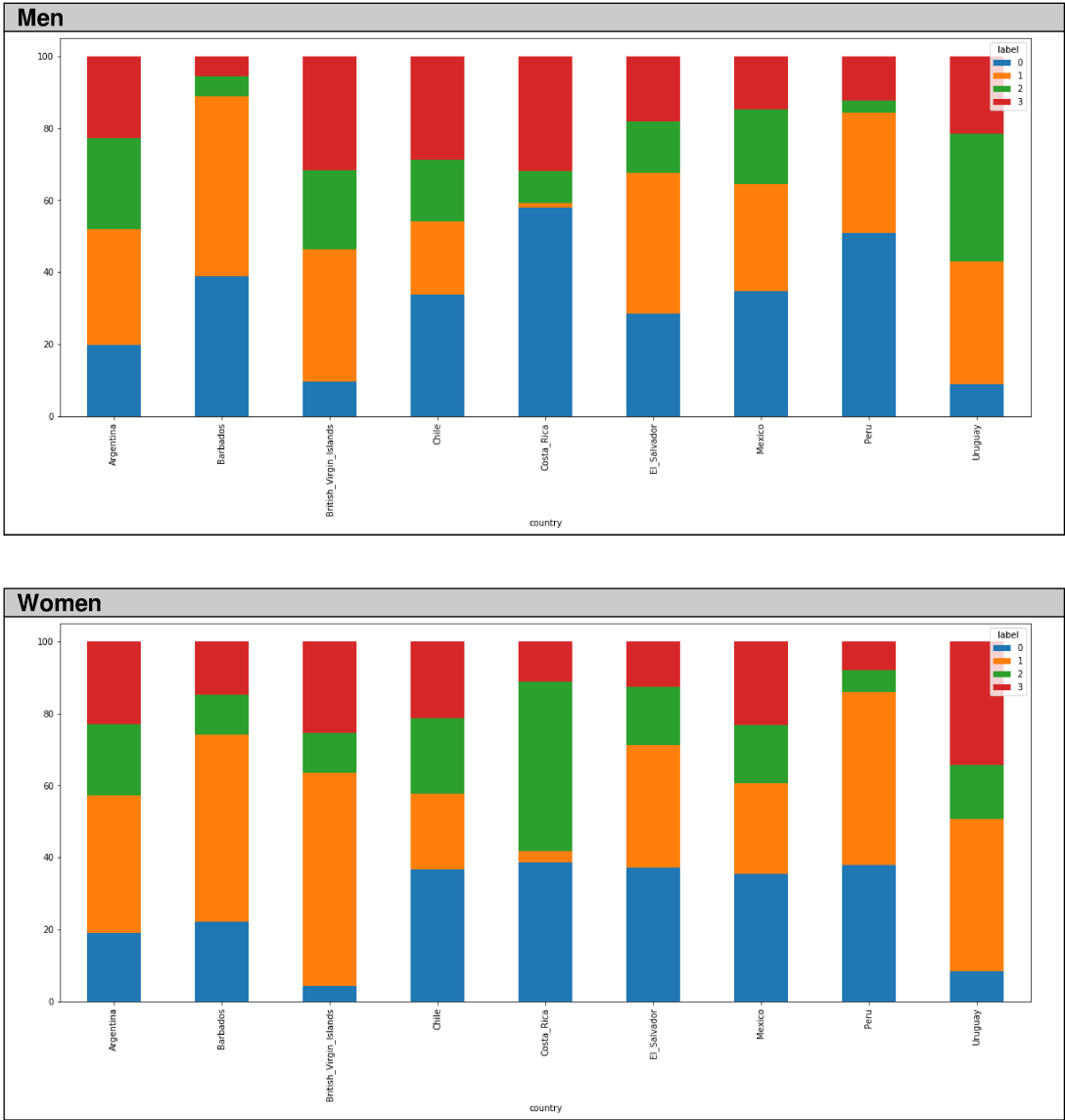

Supplement: Supplementary data [file bmjdrc-2020-001889supp006.pdf]
