# Supplementary material for: Clusters of people with type 2 diabetes in the general population: unsupervised machine learning approach using national surveys in Latin America and the Caribbean
Source: BMJ Open Diabetes Res Care. 2021 Jan 29;9(1):e001889. doi: 10.1136/bmjdrc-2020-001889 (PMC7849890; doi:10.1136/bmjdrc-2020-001889)
Supplement: Supplementary data [file bmjdrc-2020-001889supp002.pdf]

Supplementary Table 2: Jaccard analysis

	Cluster 1	Cluster 2	Cluster 3	Cluster 4
Overall	0.9763246<br>Dissolved: 0<br>Recovered: 400	0.9759331<br>Dissolved: 0<br>Recovered: 400	0.98643601<br>Dissolved: 0<br>Recovered: 400	0.9757744<br>Dissolved: 0<br>Recovered: 400
Men	0.9122498<br>Dissolved: 1<br>Recovered: 396	0.9195031<br>Dissolved: 1<br>Recovered: 399	0.91491739<br>Dissolved: 0<br>Recovered: 398	0.9091725<br>Dissolved: 1<br>Recovered: 397
Women	0.9122498<br>Dissolved: 1<br>Recovered: 396	0.9195031<br>Dissolved: 1<br>Recovered: 399	0.91491730<br>Dissolved: 0<br>Recovered: 398	0.9091725<br>Dissolved: 1<br>Recovered: 397

Carrillo-Larco RM, *et al. BMJ Open Diab Res Care* 2021; 9:e001889. doi: 10.1136/bmjdrc-2020-001889
